# Improved Transferability of Data‐Driven Damage Models Through Sample Selection Bias Correction

**DOI:** 10.1111/risa.13575

**Published:** 2020-08-24

**Authors:** Dennis Wagenaar, Tiaravanni Hermawan, Marc J. C. van den Homberg, Jeroen C. J. H. Aerts, Heidi Kreibich, Hans de Moel, Laurens M. Bouwer

**Affiliations:** ^1^ Deltares Delft The Netherlands; ^2^ Institute for Environmental Studies VU University Amsterdam The Netherlands; ^3^ 510 An initiative of the Netherlands Red Cross The Hague The Netherlands; ^4^ GFZ German Research Centre for Geosciences Potsdam Germany; ^5^ Climate Service Center Germany Helmholtz‐Zentrum Geesthacht Hamburg Germany

**Keywords:** damage modeling, disaster risk management, domain adaptation, flood risk management, loss modeling, machine learning, sample selection bias correction

## Abstract

Damage models for natural hazards are used for decision making on reducing and transferring risk. The damage estimates from these models depend on many variables and their complex sometimes nonlinear relationships with the damage. In recent years, data‐driven modeling techniques have been used to capture those relationships. The available data to build such models are often limited. Therefore, in practice it is usually necessary to transfer models to a different context. In this article, we show that this implies the samples used to build the model are often not fully representative for the situation where they need to be applied on, which leads to a “sample selection bias.” In this article, we enhance data‐driven damage models by applying methods, not previously applied to damage modeling, to correct for this bias before the machine learning (ML) models are trained. We demonstrate this with case studies on flooding in Europe, and typhoon wind damage in the Philippines. Two sample selection bias correction methods from the ML literature are applied and one of these methods is also adjusted to our problem. These three methods are combined with stochastic generation of synthetic damage data. We demonstrate that for both case studies, the sample selection bias correction techniques reduce model errors, especially for the mean bias error this reduction can be larger than 30%. The novel combination with stochastic data generation seems to enhance these techniques. This shows that sample selection bias correction methods are beneficial for damage model transfer.

## 1 INTRODUCTION

Over the last decades, both the developed and the developing world have seen an increase in the frequency and severity of hydrometeorological disasters and their impacts. Many sectors are affected and can benefit from improved models to predict these impacts, so that better decisions can be taken to reduce, retain, transfer, or absorb the risk (Van den Homberg & McQuistan, 2019). Natural hazard damage models predict the damages of a disaster given hazard characteristics such as the water depth of a flood (e.g., Merz, Kreibich, Schwarze, & Thieken, 2010) or the wind speed of a cyclone (Pielke, 2007). They are used to estimate risk from natural hazards in order to support decisions about investments in risk reduction measures. An example is their crucial role for determining the required protection levels of the dikes in the Netherlands (e.g., Kind, 2013; Van der Most, Tanczos, De Bruijn, & Wagenaar, 2014). Damage models are also increasingly used for providing impact information in early warning systems (e.g., Bachmann et al., 2016), and many national meteorological and hydrological organizations are attempting to move from hazard forecasts to impact‐based forecasts (WMO, 2015) whereby damage models are essential. Several actors, such as humanitarian organizations, can use these impact‐based forecasts to initiate early actions that reduce risks just before a hazardous event (Coughlan de Perez et al., 2015). Once the disaster has hit, similar models can be used to prioritize humanitarian aid (risk absorption) (Van den Homberg, Visser, & Van der Veen, 2017; Van der Veen, 2016; Van Lint, 2015). Damage models or so‐called catastrophe models are also applied in the insurance sector to determine premiums (Grossi & Kunreuther, 2005; Merz et al., 2010; Pielke, Landsea, Musulin, & Downton, 1999).

Traditionally, damage models often follow relatively simple approaches to estimate damages. For example, flood damage models typically relate a single variable “water depth” to resulting damage using “depth‐damage curve” (Merz et al., 2010), whereas typhoon damage models similarly relate maximum wind speed to storm damage (Pielke, 2007; Van den Homberg et al., 2017; Van Lint, 2015). However, these simple models show considerable uncertainty in their damage estimates (De Moel, Bouwer, & Aerts, 2014; Gerl, Kreibich, Franco, Marechal, & Schröter, 2016; Wagenaar, De Bruijn, Bouwer, & De Moel, 2016) and do not always perform well when they are transferred (e.g., Jongman et al., 2012). The main reason for the uncertainty is that the damage functions contain implicit assumptions about variables and processes that are not included in the model (Wagenaar et al., 2016). Examples of such variables are: flood duration, flow velocity, building construction and materials, precautionary measures, contamination of the flood water, and household size.

Nateghi, Guikema, and Quiring (2011) introduced machine learning (ML) methods to predict impacts of natural hazards (electricity outages from storms). Merz, Kreibich, and Lall (2013) used a similar approach to predict flood damages at individual building level. Since then such techniques have been applied by many authors to predict all sorts of impacts from natural hazards (Amadio et al., 2019; Ganguly, Nahar, & Hossain, 2019; Carvajal et al., 2018; Mayfield et al., 2018; Nateghi, Guikema, & Quiring, 2014; Schröter et al., 2014, 2018; Sieg, Vogel, Merz, & Kreibich, 2017; Wagenaar, de Jong, & Bouwer, 2017; Wagenaar, Lüdtke, Schröter, Bouwer, & Kreibich, 2018). These data‐driven damage models often use more than one variable to predict the damage (multivariable models). Therefore, they often perform better than traditional flood damage models (Kreibich, Müller, Schröter, & Thieken, 2017; Wagenaar et al., 2017), particularly when models are transferred (Schröter et al., 2014; Wagenaar et al., 2018). In practice, damage models are always applied in a transfer setting (Cammerer, Thieken, & Lammel, 2013). This is, for example, a model built on data or knowledge from one location applied in another location (spatial transfer), or data collected at one moment in time being applied at a different time (temporal transfer). Detailed data on flood damages are rarely recorded in a structured and consistent way and are often outdated. Some recent examples where empirical damage data were collected are described by Kienzler, Pech, Kreibich, Müller, and Thieken (2015), Poussin, Botzen, and Aerts (2014), and Molinari et al. (2014) for cases in Germany, France, and Italy, respectively.

ML methods assume that the training data to build the model consist of randomly drawn samples from the same distribution as the test samples for which the learned model needs to make predictions (Zadrozny, 2004). In a spatial and temporal transfer setting, this is often not the case. For example, damages from moderate typhoons may be used to predict the damage of an extreme typhoon. In such cases, the ML algorithms need to rely on outlier observations in the data to build the most crucial part of the model. This problem is called the “sample selection bias.” This received considerable attention in econometrics for the application to linear regression (Zadrozny, 2004). In the year 2000, Heckman (1979) received the Nobel prize in economics for developing a correction method. This “Heckman” correction, however, only applies to linear regression models. Cortes, Mohri, Riley, and Rostamizah (2008) provided two techniques to correct for this problem in case other ML methods are applied: these techniques are cluster‐based estimation (CBE) and kernel mean matching (KMM). In this article, we apply, to our knowledge for the first time, sample selection bias correction techniques (also known as domain adaptation) to damage models for natural hazards and show their potential benefits. We also introduce a variation of the CBE method that we call single variable distribution matching (SVDM), which only uses the most relevant variable.

Sample selection bias correction techniques give weights to the training data to make the most relevant samples more important during the training of the ML models. However, such techniques can result in very high weights given to single observations. In our analyses, we therefore explore a new combination of techniques where very high weights are smoothed out before they are included in the ML model. This is done by resampling the data after the sample selection bias correction with a statistical model. The resulting synthetic data are used to then train the ML models. This synthetic data generation in combination with sample selection bias correction methods is a new approach.

Sample selection bias correction techniques have never been applied to correct multivariable data‐driven models to predict the impacts of natural hazards. The objective of this research is therefore to evaluate how three sample selection correction techniques (CBE, KMM, and SVDM) reduce the sample selection bias for multivariable data‐driven damage models and improve their performance when they are transferred between different events and between different geographic locations. These methods are evaluated with and without resampling of synthetic data and with two different ML methods: artificial neural networks (ANNs) (Breiman, 2001) and random forests (RFs) (Rumelhart, Hinton, & Williams, 1986). In total, 12 different model setups are compared. These methods are applied to two different case studies where data‐driven multivariable damage models are transferred in time and space. The first case study is based on a data set with typhoon damages in the Philippines on macrolevel (municipalities). The second case study is an extension of the paper of Wagenaar et al. (2018), where multivariable microscale (buildings) flood damage models are transferred between the Netherlands and Germany. This article starts with an explanation of the methods, including an introduction to the case studies, the data and the evaluation metrics used to assess the model performance. Next, the results are presented and discussed, and finally the conclusions are presented.

## 2 METHODS AND DATA

**Fig 1:**
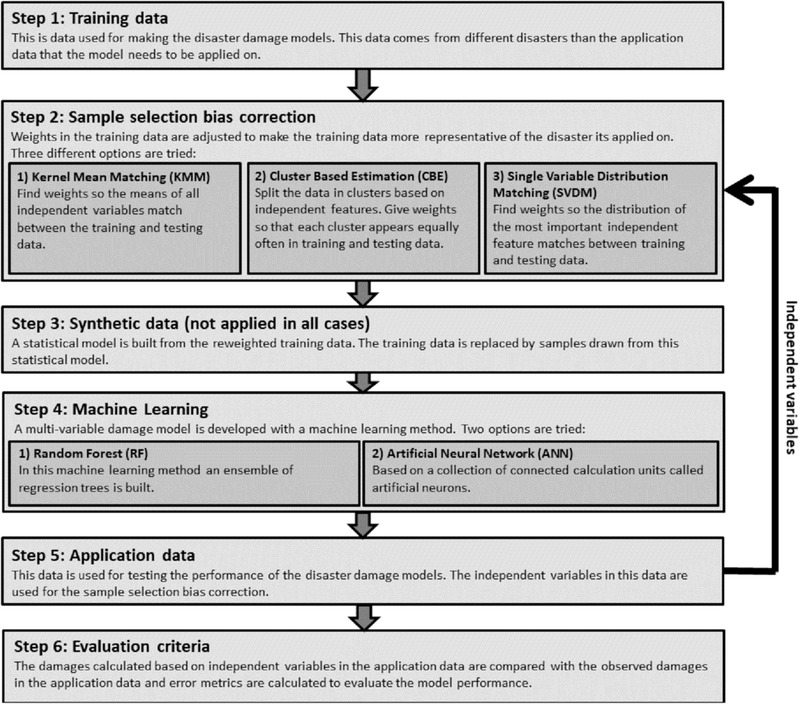
Overview of the approach for developing multivariable damage models from observational data, including the testing procedure.

Fig. 1 presents our method with six steps to improve damage estimation in transfer settings with data‐driven multivariable models based on ML techniques. The first step consists of selecting and developing training data for the damage models. These data come from different events than the application (test) data for which the model needs to predict the damages. The second step is to apply three different sample selection bias correction techniques on a training data set. The corrected training data are subsequently either directly used in two ML techniques (RFs and ANNs) to estimate damages (steps 4 and 5), or is first resampled using a statistical model (step 3). Step 3 is only applied to test the influence of generating synthetic data. The resulting damage estimates are evaluated with various error metrics (mean absolute error [MAE], mean bias error [MBE], and symmetric mean absolute percentage error [SMAPE])(step 6). This approach is applied to both case studies (flood damage and typhoon wind damage). Below, the data‐driven approaches are further described (Section 2.1), the case studies are introduced (Section 2.2), the specific model setup to apply the data‐driven approaches to the case studies is shown (Section 2.3), and finally the evaluation metrics are specified (Section 2.4).

### 2.1 Data‐Driven Methods

#### 2.1.1 Sample Selection Bias Correction

##### 2.1.1.1 Kernel mean matching

KMM (Cortes et al., 2008) assigns a set of weights to the training data, so that the mean of each variable in the training data becomes as close as possible to the mean of each variable in the test data. This is called a covariate shift. These weights are determined with an optimization algorithm. The optimization problem is shown in formula 1 (Cortes et al., 2008). The data need to be normalized before applying the KMM algorithm.

(1) $$\min_{\gamma }\;G\;\left(\gamma \right)=\left|\left|\frac{1}{n_{tr}}\sum_{i=1}^{n_{tr}}\gamma \Phi \left(x_{i}^{tr}\right)-\frac{1}{n_{te}}\sum_{i=1}^{n_{te}}\Phi \left(x_{i}^{te}\right)\right|\right|,$$

where γ is the vector with weights that is determined by the optimization algorithm, G(γ) is the distance between the means of the weighted training data and the testing data that is minimized, $x_{i}^{tr}$are the independent variables only of the training data, $x_{i}^{te}$ are the independent variables only of the test data, *n* is the number of observations in the training or test data, and Φ(x) is the kernel function that maps *x* to a reproducing kernel Hilbert space (Berlinet & Thomas‐Agnan, 2004). A weakness of KMM is that it gives equal importance to all independent variables. Another weakness of KMM is that the algorithm only matches the mean but not the entire distribution between training and test data. There are many different solutions to get to a matching mean. Some might not lead to a better match of the entire distribution, for example, when large weights on error prone outliers are applied to shift the mean. Since the damage models are sensitive to extreme values, it would be desirable that the sample selection bias correction method leads to a better match of the entire distribution.

##### 2.1.1.2 Cluster‐based estimation

In CBE, the entire data set (training and test data) is first split into several clusters. These clusters are made by combining the independent variables of the training and test data and then applying an unsupervised learning algorithm to find clusters of observations that lie relatively close together. After the clusters are identified, both the training and test data are split into these clusters. The weights are then determined in such a way that each weighted cluster appears as frequently in the training data as it appears in the test data. See the following formula:

(2) $$CW_{x}=\frac{\frac{N_{x,\mathrm{test}}}{N_{\mathrm{test}}}}{\frac{N_{x,\mathrm{train}}}{N_{\mathrm{train}}}},$$

where *CW_x_* is the cluster weight to be applied on each sample in the training data that belong to the specific cluster. *N_x,_*_test_ is the number of samples in the test data that belong to that cluster, *N*_test_ is the total number of samples within the test data. *N_x,_*_train_ and *N*_train_ are the same but for the training data.

The unsupervised learning algorithm *k*‐means clustering is applied. This algorithm splits the data in *K* clusters based on the nearest means by placing *K* points in the spectrum of the data. It then clusters each data point based on which of the *K* points it is most close to (Kanungo, Mount, Piatko, Silverman, & Wu, 2002). The algorithm then optimizes the position of the *K* points in such a way that the total distance of all data points to the locations of the *K* points is minimized. The data need to be normalized before applying the algorithm.

Just as the KMM method, the disadvantage of CBE is that all variables are equally important, while in fact the variables differ in their importance for predicting the damage. For example, wind speed is often a more important variable than the economic growth of a municipality, in the case of wind damage estimation. Since all variables are assumed to have the same importance in the clustering, this may lead to clusters that are not particularly relevant for reducing the sample selection bias.

##### 2.1.1.3 Single variable distribution matching

The CBE method is normally trying to match the distributions of all different variables. Some of these variables are, however, less important for the damage estimation than others. The CBE method is unaware of this difference in importance and will only try to match all available variables with equal importance. Matching the distributions for each variable perfectly is not possible on such small data sets, so compromises are made. These compromises reduce the quality of the match in the more important variables and therefore may reduce the model performance compared with a method that focusses on the most important variable.

Therefore, we introduce a special configuration of the CBE, which we call SVDM. This method makes use of the expert knowledge on the most important variable for the damage model. This works by just supplying the CBE method with the most important variable, such as water depth for floods or the wind speed for typhoons.

A disadvantage of adjusting for the most important variable only is that sometimes a transfer needs to be made over multiple variables. For example, a transfer in both building styles and water depth would be impossible with this approach. It is, however, possible to optimize this method by using several important variables rather than only the most important one. Such configurations are not explored in this research and rather only the two most extreme configurations are applied: that is, all variables in common CBE or only the most important variable in the case of SVDM.

#### 2.1.2 Synthetic Data Generation

The sample selection bias correction methods sometimes generate high weights for specific observations, for instance when one observational value is 30 times more important than another. Generating synthetic training data by resampling can create new data with similar statistical characteristics to the weighted training data. This results in many data points similar to the observation with the high weight rather than one specific point with a very high weight. This can be done with synthetic data generation techniques that are applied for example to meteorological or river discharges data (Diermanse, Carroll, Beckers, Ayre, & Schuurmans, 2012).

This synthetic data generation approach to smooth out the high weights has been inspired by a similar method called synthetic minority oversampling technique (SMOTE) (Chawla, Bowyer, Hall, & Kegelmeyer, 2002). This technique helps to correct imbalanced training data in classification problems, for instance, when rare observations in the training data need to be predicted.

Synthetic data are generated by building a statistical model that represents the sample selection bias corrected training data. From this statistical model, new data points are sampled. This procedure can be summarized as follows:

- A Kendall's rank correlation matrix (*T*) is derived from the training data. The matrix is a square matrix with the size of the number of variables.
- A matrix *P* is derived through Cholesky decomposition, in which *P* × *P* – 1 = sin(phi *T*/2) (Fang, Fang, & Kotz, 2002).
- For each variable, sample values with the standard normal distribution function are generated using its mean and standard deviation.
- Correlation is introduced between these individual samples. Such correlated samples are calculated based on multiplication between the transpose of matrix *P* and the sample values for each variable.
- To go from the normally distributed to the originally observed distributions in the training data, an inverse transformation is applied to the normalized correlated sample based on the variable's empirical distribution.

#### 2.1.3 ML Techniques

ML is a field of artificial intelligence that provides computer systems the ability to learn from data without being explicitly programmed. ML algorithms are classified into (i) supervised learning, (ii) unsupervised learning, and (iii) reinforcement learning.

This study focuses on the application of supervised learning algorithms (Praveena & Jaiganesh, 2017) to build models that can explain the complex relationships between damages and the variables that can explain damages, such as water depth or wind speed. We applied RF and ANNs in this study. RFs are chosen because they have a good track record in damage modeling (e.g., Amadio et al., 2019; Ganguly et al., 2019; Schröter et al., 2018; Sieg et al., 2017; Wagenaar et al., 2017; Wagenaar et al., 2018), ANNs have also been used before in flood damage models (Amadio et al., 2019; Ganguly et al., 2019), and in this study they were selected because of their ability to extrapolate and at the same time find complex nonlinear relationships. Table I provides a comparison between the ML methods. The *K*‐means unsupervised learning algorithm is applied within the CBE sample selection bias correction technique.

**Table I:** Comparison of the Random Forest (RF) and Artificial Neural Networks (ANN) Machine Learning Methods

RF	ANN	Reference
Capture nonlinear relationships		Nawar and Mouazen (2017)
Overfitting may occur when too many splits in a tree are made	Overfitting may occur when too many hidden layers are included	Ahmad, Mourshed, and Rezgui (2017), Breiman (2001)
Has few tuning parameters, which are often insensitive	Has more tuning parameters	Ahmad, Mourshed, et al. (2017), Breiman (2001)
When applied to the same data set, typically, faster to train	When applied to the same data set, typically, slower to train	Ahmad, Hippolyte, Mourshed, and Rezgui (2017)
Cannot extrapolate	Can theoretically extrapolate	Tyralis et al. (2019)
Provides probabilistic predictions	Provides deterministic predictions	

##### 2.1.3.1 Random forest

RF, an ML method developed by Breiman (2001), has been used in flood damage modeling (e.g., Amadio et al., 2019; Ganguly et al., 2019; Schröter et al., 2018; Sieg et al., 2017; Wagenaar et al., 2017; Wagenaar et al., 2018). RFs are ensembles of regression trees where the data for each tree are generated by a bootstrapping resampling method. In each tree, branches are formed by splitting the data set based on binary recursive partitioning, for instance, a partition of data based on whether the average wind speed is higher than a certain value. The RF algorithm does not use all explanatory variables for each tree, but it seeks the best splits within a limited number of sampled explanatory variables. The number of sampled features is the square root of the total number of features in the data sets. The best splits refer to regression trees that split the training data in such a way that there is as little variation as possible within the resulting leaves. The predicted value for the entire RF is the mean value of the predictions from each regression tree.

A disadvantage of an RF is that they can never make a prediction that is higher than the values seen in the training data, hence it cannot extrapolate (Tyralis, Papacharalampous, & Langousis, 2019). This is because each regression tree has a set number of leaves. When making a new prediction it will place the prediction in an existing leaf. It cannot create a new leaf with a higher damage value. In a damage model transfer setting, this inability to extrapolate can be a disadvantage as extrapolation is sometimes required. An advantage of RFs is that they can make probabilistic predictions, which is, however, not used in this article.

##### 2.1.3.2 Artificial neural network

An ANN is an ML framework inspired by how the human brain processes information (Hassoun, 1995). It was first introduced by Rumelhart et al. (1986), ANNs gain knowledge through learning the relationships between variables in a data set without any given information about the system. The model built based on ANNs consists of several (hidden) layers of neuron‐like processing units connected with each other. Each neuron is connected to all other neurons in the layer before it and after it. The connections work through coefficients that weigh each value that comes through the neuron. The coefficients of the neurons are determined with an optimization algorithm that minimizes the error on the training data set. A strength of ANNs is that they can simulate complex nonlinear patterns. Larger ANNs with more neurons can represent more complex nonlinear patterns but are also more prone to match the training data so well that it works poorly on new cases (overfitting). The model built in this study is based on a multilayer perceptron (MLP) ANN, which consists of an input layer, two hidden layers, and an output layer (prediction). For transferring multivariable damage models, ANNs may have an advantage over RFs in that they can extrapolate. In an ANN inputs are multiplied with coefficients. When the input value in the test data (e.g., water depth) is larger than the inputs in the training data, the predicted value will be also larger. A general disadvantage of ANNs is that their predictions are deterministic and hence less suitable for applications that would benefit from having probabilistic estimates.

### 2.2 Case Studies

A case study approach was used to quantitatively assess the improvement of the spatial and temporal transferability of damage models based on an ANN or an RF upon applying the three sample selection bias correction methods. Two case studies were used to allow a deeper insight into the application of damage models at two different spatial scales: macrolevel (municipalities) and microlevel (buildings).

Macrolevel damage models predict the damage based on the aggregated data within one administrative boundary (e.g., village, district). This detailed level is sufficient for many applications and the data are easier to collect. For the macrolevel, a case study with typhoons in the Philippines on municipality level was adopted. The models in this article are an extension of macrolevel data‐driven multivariable models that were developed to support humanitarian aid organizations with the prioritization for distributing humanitarian aid after or just before a typhoon. The models aim to provide timely and unbiased information after a disaster, which are often difficult to obtain using current practices (field surveys).

Microlevel damage models, on the other hand, predict the damage on disaggregated level (e.g., per building). Microlevel models are often used for disasters that require a detailed spatial resolution such as in our case for damage from fluvial floods in Europe. Such level of detail is required in insurance applications when risk premiums need to be determined per building, or for flood mitigation policies when measures on building level are considered. Even though for many such applications the results are later aggregated, the calculations are often done on microlevel because macromodels can lead to considerable spatial uncertainty (Wagenaar et al., 2016).

The data used have been selected after an assessment of the data quality on different attributes, that is, timeliness, source (reliability), accuracy, and granularity/spatial coverage (Van den Homberg, Monné, & Spruit, 2018) as will be explained for each case study. Obviously, the data for both the independent and dependent variables need to be available at the same granularity and spatial coverage. Table II summarizes the characteristics of the two case studies. In both cases, the data are always used in a transfer setting. It means the data are applied on an event or a location that was not part of the training data.

**Table II:** Characterization of Case Studies

Case Study	Level	Event	Data Set	Applications	Transferability (Time and Space)	Data Preparation and Machine Learning
Typhoons, The Philippines	Macro, Relative damage at municipality level	12 typhoons 2012–2016	1,600 records of percentage totally destroyed buildings in municipalities	Area prioritization on distribution of humanitarian aid	The use of data from historical typhoons to predict the damage of another typhoon. Mostly transfer over time.	Sample correction bias methods Synthetic data generation (3,000–10,000 samples) cross validation between events based on ANN/RF
Fluvial floods, The Netherlands and Germany	Micro, Relative (content and structural) damage at building level.	1 flood event 1993	The Netherlands: 4,398 monetary residential damages.	Insurance, risk reduction and mitigation, cost–benefit analysis	The use of Dutch flood damage data to predict the damage from floods in Germany. Both transfer in time and space.	Sample correction bias methods Synthetic data generation (3,000–10,000 samples)
		6 flood events 2002 –2013	Germany: 895 monetary residential damages			Transferring Dutch model to Germany based on ANN/RF

Apart from the spatial scale, the cases use different types of variables, damage mechanisms and type of transfer. The macro case study has more vulnerability type variables such as poverty and building materials, and has in some cases more damage mechanisms, such as floods due to a storm surge caused by the typhoon. The transfer for the macromodel was over time since all data come from the same larger study area. In the micromodel there is both a time (different events) and space transfer (between the Netherlands and Germany). These large differences are an ideal test to see whether the sample selection bias correction techniques work under different circumstances.

#### 2.2.1 Macrolevel Model: Philippines Typhoons

On average around 20 typhoons strike the Philippines annually and more than half of them make landfall in the country (Reliefweb, 2017). Typhoon Haiyan (local name Yolanda), which hit the Philippines in 2013, is considered one of the strongest tropical cyclones ever recorded. The fatalities caused by the typhoon amounted to about 6,000 people, around 14 million people were affected and more than 1 million houses were damaged (World Bank, 2014).

510, an initiative of the Netherlands Red Cross collated the typhoon damage data in this case study through desk research and in‐country visits of key stakeholders. The purpose of collating these data is to populate 510's community risk assessment dashboard and to develop a model that can be used to predict the areas with the highest damage either just before the disaster to trigger early action or just after the disaster to improve efficiency in the aid distribution process.

##### 2.2.1.1 Data

Data have been gathered on 12 typhoons in the Philippines at the municipality level. The median number of households in a municipality is around 6,600. The data set contains about 1,600 damage records, with 40% of those damage records corresponding to the two typhoons that cover the largest extent. This does not necessarily mean that they have the largest aggregate damages.

The vulnerability and exposure variables in a municipality are the same for all typhoons while the hazard features are specific to a typhoon. The vulnerability and exposure may have changed over time in the period from 2012 to 2016, due to, for example, population growth and land use change. These changes, however, are typically relatively slow. Recovery efforts are an exception because damages could be lower in an area that was recently affected and has not recovered yet. This can be a source of variation in the data but is expected to be limited.

The data set collected by the Red Cross consists of more than 40 variables from which damage is to be predicted. Table III presents the variables that were used to build the damage models for the macro case study. It is essential to have data on these independent variables with national spatial coverage and at the same administrative levels. The municipality level was chosen as the smallest geographic level because this is the lowest resolution on which all the data are available.

**Table III:** Variables Available for the Macro Case Study

Variable name	Unit	Source(s)	Remarks (Model Scale)
Completely destroyed buildings (damage)	%	National DRR and Management Council (NDRRCM)	Percentage of the houses that are entirely destroyed and unfit for habitation or without any remaining structural features. Data collected for Emergency Shelter Assistance program (DSWD, 2019)
Average wind speed	mph	Tropical Storm Risk (UCL, 2018)	Maximum three seconds sustained gust speed over the event in the particular municipality. Every municipality has a unique wind speed calculated based on the forecasted maximum wind speed on the track and the method from Holland (1980) to calculate it for the specific municipality.
Accumulated rainfall	mm	Meteorological data from Global Precipitation Measurement (GPM) (Huffman et al., 2015)	Total accumulated rainfall during the typhoons period from satellite data (Huffman et al., 2015).
Number of households		Philippines National Census	2010 data, unique value available for each municipality in the country.
Population density	people/km^2^	Philippines National Census	2010 data unique value available for each municipality in the country.
Area	km^2^	GIS analysis	Area within the official municipality boundaries.
Elevation (average and weighted on population)	m	STRM (NASA, 2013)	30‐Meter Elevation Data
Slope	m/m	SRTM (NASA, 2013)	Based on QGIS (2020) applied to 30‐Meter Elevation Data.
Roof types (wood, iron, straw, concrete, semiconcrete) in an area	%	Philippines National Census	Based on 2008 data, unique value available for each municipality in the country.
Wall types (concrete, makeshift, wood, concrete, iron, bamboo) in an area	%	Philippines National Census	Based on 2008 data, unique value available for each municipality in the country.
Population under poverty line	%	Philippines Statistics Authority	Available per province, each municipality has the province value.
Length coastline	m	GIS analysis	Based on official municipality boundaries.
Ruggedness	m	SRTM (NASA, 2013)	This is the Terrain Ruggedness Index, defined as the mean difference between a central pixel and its surrounding cells, calculated on 30‐m SRTM elevation data with the QGIS (2020)
Population living 500, 1,000, and 1,500 m from the coast	%	WorldPop (Gaughan, Stevens, Linard, Jia, & Tatem, 2013)	Based on a GIS analysis combined with WorldPop data (worldpop.org.uk)
Economic growth	%	Philippines Statistics Authority	Annual growth for the year 2018. Available per province, each municipality has the province value.
Population growth	%	Philippines Statistics Authority	Annual growth for the year 2018. Available per province, each municipality has the province value.

##### 2.2.1.2 Model setup and validation

The article proposes to build data‐driven damage models that can be part of a model set‐up used for operational purposes on newly predicted events. The evaluation can be carried out by using one of the observed typhoons as test data and use the rest as training data. The damage caused by a historical typhoon is predicted by a model built based on the data from the other 11 typhoons. As there are data about 12 typhoons recorded in the data set, 12 prediction models were built in total with each typhoon serving once as the test data for which the model is then tested.

Data‐driven damage models were developed to predict the percentage of completely destroyed buildings in an affected municipality based on the variables shown in Table III. The most interesting aspect about the damage data is that the average of damage varies between the 12 typhoons. The average value over all typhoons is 6% of the buildings completely destroyed, which is nearly six times smaller than the average for typhoon Haiyan.

**Fig 2:**
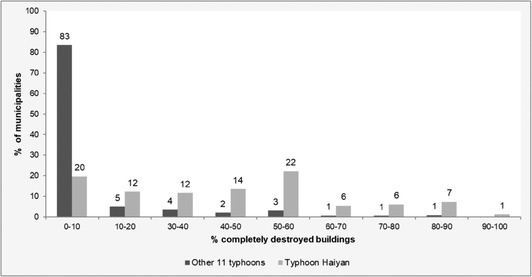
The distribution of completely destroyed houses per municipality for the Haiyan typhoon compared to the other typhoons that were used to build a model for Haiyan.

From Fig. 2, it can be seen that the distribution of the damage to buildings caused by typhoon Haiyan is much higher than for the other 11 typhoons. This indicates that the damage data from the other 11 typhoons that are used to build the prediction model for Haiyan are not fully representative for this typhoon and hence a major model transfer is required that includes extrapolation. This is a typical example where advances in the transferability of models may improve damage predictions.

#### 2.2.2 Microlevel Model: European Flood Damage Models

Damage data and independent variables for the microlevel case study were selected for six past river flood events in Germany between 2002 and 2013 and for one river flood event in the Netherlands in 1993. These data have been used for several data‐driven models in the past (Wagenaar et al., 2017, Wagenaar et al., 2018, Schröter et al., 2014; 2018, Merz et al., 2013. In the current microlevel case study, a multivariable flood damage model made based on Dutch data is transferred to Germany. The same model transfer was done in the paper by Wagenaar et al. (2018), which showed that this model transfer could potentially be improved, as it was the model with the lowest performance, owing to the low variability of the damage data in the 1993 flood event in the Netherlands. The expectation therefore is that the model can be improved by correcting for the known sample selection bias. The flood damage model predicts the relative damage on building level based on the variables shown in Table IV.

**Table IV:** Variables used in the Microlevel Case Study (for More Information, see Wagenaar et al., 2018)

Variable Name	Unit	Source Dutch Data Set	Source German Data Set	Remark
Relative building damage	–	Inspection and building value estimate	Phone interview	Relative to potential damage.
Relative content damage	–	Inspection and content value estimate	Phone interview	Relative to potential damage.
Water depth relative to ground floor	m	Inspection	Phone interview	
Building type		Inspection	Phone interview	Two types available, attached or unattached.
Footprint area building	m^2^	Cadastre	Phone interview	
Water depth relative to DEM	m	Model		For German data equal to water depth relative to floor.
Basement		Inspection	Phone interview	
Household size	#	Inspection	Phone interview	
Flow velocity	m/s	Model	Phone interview	For German data estimated from score
Building age	Year	Cadastre	Phone interview	
Floor area for living	m^2^	Cadastre	Phone interview	
Flood duration	hour	Hydro‐dynamic Model	Phone interview	
Return period	year	Statistical model	Statistical model	Definition in: Wagenaar et al. (2018)

##### 2.2.2.1 Data

The Dutch training data in this case study are derived from observed flood damages after the 1993 flood in the Meuse River in Limburg reported in WL Delft (1994), supplemented with data on building and flood characteristics documented in Wagenaar et al. (2017).

The model is applied to predict the damage from six different flood events in Germany. Damage from these floods including relevant building and flood characteristics were collected using phone interviews (Thieken, Kreibich, Müller, & Merz, 2007, Kreibich et al., 2017). The German data set contains a wide range of values for the different flood characteristics and circumstances (Kreibich et al., 2011, Kienzler et al., 2015), the Dutch data are on the other hand more homogenous because they are based on only one flood event (Wagenaar et al., 2018).

##### 2.2.2.2 Model setup and validation

There are 895 damage observations from the German data that can be used to test the model developed based on the 4,398 damage observations from the Dutch data. To reduce the randomness in the predictions due to the specific selection of training samples, bootstrapping is applied (Efron & Tibshirani, 1993). In bootstrapping, a random sample of the training data is taken to train the model, and then a random sample of the test data is taken to test the model. Samples are taken with replacement. This is repeated several times, so that many models are trained and tested on such subsets of the data. For each bootstrap run, 4,000 training samples from the Dutch data and 350 testing samples from the German data were randomly picked. Bootstrapping reduces the chance that a difference between the two samples is due to randomness rather than because of an improvement in the prediction method. For the RF, 100 bootstrap samples were taken. On the other hand, only 20 bootstrap samples were taken for ANN due to the greater calculation time. Less samples were taken for the ANNs, as differences between the calculated errors were shown to be minor, while the calculation time was much longer for the ANN than for RF.

### 2.3 Model Parameters

Damage models built based on RF and ANNs have been developed using the Python 2.7 library “Sci‐Kit learn” (Pedregosa et al., 2011). For the damage model based on RFs, 100 regression trees were grown. More regression trees need more computation time but also typically give better results. This improvement from adding more trees becomes negligible after a certain number of trees. For this study, the same number of trees is applied as in Wagenaar et al. (2018) and the model errors could not be reduced by adding more than 100 trees. The number of splits and minimum number of observations per leaf were optimized. For the prediction model based on ANN, learning rates and number of neurons in the first hidden layer were optimized. The number of neurons in the second hidden layer was fixed to be half of the neurons in the first layer.

This optimization was carried out by randomly splitting the data set into 60:40 for the training and test set. The tuning of the parameters for both models was carried out to result in the smallest MAE on validation data that did not involve a model transfer (splitting the training data randomly).

The CBE and SVDM methods have one parameter to tune: the number of clusters used. This was chosen to be 12 clusters for both case studies. The KMM method has only one parameter to be optimized also: the kernel to be used. Linear kernel was chosen because of its simplicity. For SVDM, the most important variable to predict the damage chosen was wind speed for the macromodel and water depth for the micromodel. Both variables are widely used in single variable damage models (e.g., Pielke, 2007; Merz et al., 2010; Gerl et al., 2016). Furthermore, the feature importance analysis carried out within the RF confirms this choice.

For the synthetic data generation, the number of synthetic data points to be generated can be optimized. More synthetic data points generated generally gives better results, but after a specific point they do not considerably affect the results anymore. For the macromodel, the number of synthetic data points to be generated is always twice the weight of the training set after the sample selection bias correction methods are applied. This is based on a minimum weight of one, so the sample selection bias correction increases the number of data points. This typically turns out to be between 3,000 and 10,000 synthetic data points. For the micromodel, a simplified approach was applied with a fixed number of samples because the training set is always the same size, this fixed number of samples is 5,000. The number of samples to be taken was estimated based on increasing the number of samples until the evaluation metrics would no longer improve.

### 2.4 Evaluation Metrics

To evaluate the model performance, three different error metrics were used: MAE, MBE, and SMAPE. Table V shows the formulas for the different evaluation criteria.

**Table V:** Criteria Applied to Evaluate the Model Performance

Evaluation Criteria	Formula
Mean absolute error (MAE)	$MAE\;=\frac{1}{N}\;\sum \;\left|RL_{\mathrm{sim},n}-RL_{\mathrm{obs},n}\right|$
Mean bias error (MBE)	$MBE\;=\frac{1}{N}\;\sum \;RL_{\mathrm{sim},n}-RL_{\mathrm{obs},n}$
Symmetric mean absolute percentage error (SMAPE)	$SMAPE\;=\frac{\sum |\;RL_{\mathrm{sim},n}-RL_{\mathrm{obs},n}|}{\sum |\;RL_{\mathrm{sim},n}\left|+\right|RL_{\mathrm{obs},n}|}\;$

The MAE is suitable to evaluate the accuracy for individual predictions. This is important when the individual model results need to be applied, for example, for insurance or for macrolevel models. The MBE shows whether there is a bias in the model, for instance, whether it consistently makes over or underestimations. This is important when the aggregated results are used. For example, in a micromodel used for a cost–benefit analysis for an infrastructure investment, only the total sum of all predictions is important rather than individual prediction per building. In such a case, the MBE is the most important evaluation criteria. The SMAPE is used in the same manner as the MAE but is a relative error metric. This allows to compare the errors of different order of magnitude events. For example, some models have predicted damages in the order of 50–80% while others have a maximum of 20%. A 20‐percentage‐point error on a damage of 80% is much lower relatively, than a 20‐percentage‐point error on a damage of 10%.

For the macromodel, the errors were evaluated for 12 different typhoons. Then the weighted mean of their errors was calculated. The weights were assigned based on the number of predicted damages in each model. For the MBE, the absolute values are taken before the mean is calculated over the 12 events. This is done in order to ensure that a positive bias in one test cannot cancel out a negative bias in another test. Consequently, all bias errors are positive. To obtain the mean that represents the quality of the 12 models, the criteria to evaluate errors should be independent from the extent of the damage they predicted. SMAPE is particularly useful for this case study, as the errors for different models are compared with each other. For the micro case, the variation between the damage cases is less extreme and therefore a SMAPE approach is not necessary.

In this article, no evaluation metrics are applied to validate the quality of the probabilistic estimates of the RF and to see whether these probabilistic estimates improve because of sample selection bias correction methods. This is not done because ANNs are not able to make such probabilistic predictions. This could be a topic for future research.

## 3 RESULTS AND DISCUSSION

Table VI compares the performance of the predictions of the different ML models as measured by the evaluation metrics described in Section 2.4. It is apparent from the highlighted numbers in this table that the best performing models in both case studies and for all evaluation criteria always have some form of sample selection bias correction included. Furthermore, on the basis of MBE evaluation criteria, all sample selection bias correction methods always outperform the reference models. The improvements on the MBE metric can be as large as 85% (e.g., MBE content damage), where many different sample selection bias correction methods result in large improvements. It is promising that the sample selection bias correction methods lead to improvements in both case studies, despite the large differences between the phenomena and data in the case studies, as discussed in Section 2.2.

**Table VI:** Performance of Different Models for Both the Micro and Macro Case Studies

			Macromodel			Micromodel			
Methods			Damaged Buildings (%)			Building Damage		Content Damage	
Machine Learning Methods	Sample Selection Bias Correction	Synthetic Data generation	MAE	MBE	SMAPE	MAE	MBE	MAE	MBE
RF			5.53	3.86	0.672	0.100	0.089	0.213	0.207
ANN			6.29	4.15	0.668	0.104	0.097	0.218	0.212
RF	KMM		5.27	3.48	0.618	0.099	0.082	0.211	0.202
ANN	KMM		5.78	3.25	0.674	0.109	**0.014**	0.204	**0.081**
RF	CBE		5.06	3.31	0.613	0.098	0.079	0.209	0.198
ANN	CBE		5.16	**2.53**	0.664	0.096	0.064	0.198	0.169
RF	SVDM		5.25	3.45	0.617	0.099	0.034	0.196	0.111
ANN	SVDM		5.69	3.53	0.661	0.097	0.085	0.211	0.205
RF	KMM	SD	4.69	3.29	**0.608**	0.095	0.065	0.198	0.171
ANN	KMM	SD	5.89	2.73	0.674	0.112	0.030	0.210	0.099
RF	CBE	SD	4.90	3.53	0.627	0.098	0.079	0.206	0.192
ANN	CBE	SD	5.63	3.40	0.672	**0.095**	0.055	0.195	0.153
RF	SVDM	SD	**4.43**	3.29	0.613	0.095	0.076	0.206	0.198
ANN	SVDM	SD	5.71	3.69	0.667	0.101	0.038	**0.194**	0.100

*Note*: The best performing model setup is made gray and bold. The second‐ and third‐best performing methods are made gray.

For the MAE metric, the results are a bit more varied. For the micromodel, the improvements are minor. On the other hand, every sample selection bias correction method provides improvements for the macromodel on the MAE criteria. The improvements for the SMAPE are, however, much smaller and are more in line with the improvements seen on the MAE for the micromodel. Some sample selection bias correction methods are also not better than the reference models without sample selection bias correction for the SMAPE. The performance on the MAE for the macromodel is mostly based on the model performance on the extreme observations, because these observations have large errors, improving them has a relatively large impact on the MAE. For the SMAPE error metric the large and small damage observations have a more equal weight in the error metric calculation. The sample selection bias correction methods therefore seem to be most relevant to predict outlier observations. These results seem to be consistent with the general idea that the sample selection bias correction is mostly suitable for extreme observations, which is very relevant for some of the applications of damage models.

In theory, these techniques should not work in a situation without a model transfer because there should not be any bias in the data when the training and test data come from the same source (i.e., same variable distributions). The weights calculated by the sample selection bias correction methods should in that case be close to one and therefore the methods do not correct for anything. To test this, the best performing sample selection bias correction methods were also applied to settings without a model transfer. For the micromodel, independent test data come from the same source as the training data (Dutch data). For the macromodel, all observations are put together and then split into training and test data. In this setting, the sample selection bias correction methods had hardly any influence on the results for the macromodel (data not shown). A reduction was seen only in the MBE on the micromodel, but without a model transfer this MBE is negligible (close to zero). Therefore, the reduction is very minimal in absolute terms.

The sample selection bias correction methods lead to a larger reduction in the MBE in combination with the ANN methods then in combination with the RF methods. Without sample selection bias correction methods, the ANN model performs less well than the RF model. This occurs consistently in both the micro and the macro case. The reason for this is not entirely clear, but we speculate that this could be due to the sensitivity of the different ML methods to the data.

### 3.1 Macro Case Study

**Fig 3:**
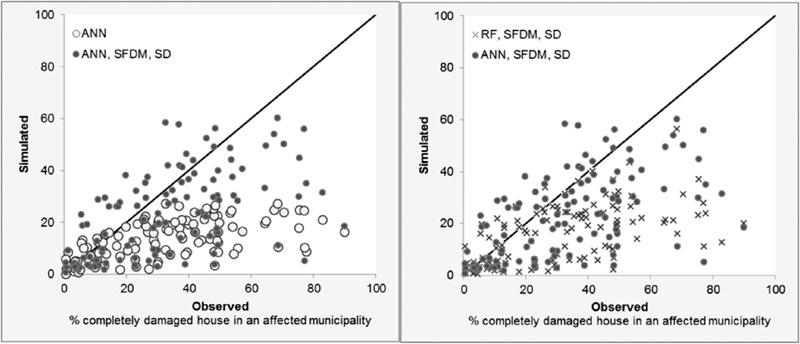
The performance per municipality for the model to predict damages for the Haiyan typhoon. Left: A comparison of the ANN method with and without sample selection bias correction and synthetic data generation. Right: A comparison of the RF and ANN methods with sample selection bias correction and synthetic data generation.

For the Philippines case study, sample selection bias correction methods have considerably improved predictions from the 12 damage models. Fig. 3 (left) visualizes an example of the improvement in predictions for the extreme typhoon Haiyan after employing the SVDM method in combination with synthetic data generation to the ANN. It shows that without sample selection bias correction the model consistently underestimates the damage as all estimates are below 30%. After implementing the sample selection bias correction method, this consistent underestimation is largely solved and damages are predicted up to 60%, as they were also observed for typhoon Haiyan.

Fig. 3 (right) provides an insight on how the different ML methods result in varying improvements. It can be seen from the figure that the ANN model results in more accurate predictions for the Haiyan typhoon compared to the RF model after the sample selection bias correction methods are applied. The results also further support the theory that a model built with an ANN is better able to predict the damage by extrapolation, compared to the RF model.

Table VI shows that the predictions from 12 models built using RF as basis ML method provide the smallest errors on average. This implies that most of damage caused by other typhoons than an extreme typhoon such as Haiyan can be better predicted by an RF that can only interpolate and not extrapolate. This makes sense because the extrapolating capacity of ANN is not required for most of the data points, apart from data points of extreme typhoons.

A possible explanation for why the ANN models perform worse for average model results than the RF models is that RF works better on a relatively small data sets. Another likely explanation is that the ANN model is quite sensitive for parameter tuning while the RF model is not. The procedure for tuning the parameters could be improved. The tuning should not be carried out for all models at once based on the randomly split data (See section 3.3), but for each of the 12 models separately. The tuning of parameters that result in the smallest weighted mean error for the 12 models together then should be applied to all the 12 damage prediction models to be evaluated.

In general, the macro case study is limited by the lack of information on exposure and vulnerability variables. Adding more variables could be helpful. Also, the data on the explanatory variables were the same for all events regardless of the year in which the typhoon hit. Over time these characteristics may have undergone change, requiring changes in the variables. For example, houses might have been built back better after a typhoon with different materials. In particular, locally this is expected to lead to some error, for instance, when large damages have occurred recently and people have responded by abandonment or much stronger building construction. These errors are, however, expected to have a negligible effect on the aggregated results of this case study.

### 3.2 Micro Case Study

Sample selection bias correction methods have reduced the MBE for all cases in the micromodel case study. In 4 of 12 cases this reduction is even larger than 50%. The MBE is the most relevant metric when the aggregated results of micromodels are used. The MAE improvements for the micromodel are rather small but in line with the SMAPE improvements of the macromodel. This is probably because outliers have a smaller influence on the aggregated MAE metric for the micromodel than for the macromodel in which large differences between damages were presented. Another possible explanation is the difference in data quality of the micromodel. The macromodel consists of municipality averages while the micromodel has values per building. The average values per municipality can correct overestimations and underestimations and hence the aleatory uncertainty is reduced. For individual building values, however, aleatory uncertainty is very high, and no such evening out of errors by averaging exists. This aleatory uncertainty cannot be reduced by sample selection bias correction methods and therefore the reductions in MAE are smaller in the micromodel.

### 3.3 Performance of New Sample Selection Bias Correction Methods

In this article, two innovations in sample selection bias corrections were introduced: using a single variable correction in the CBE method (SVDM method) and synthetic data generation. These innovations were compared to two other correction methods (KMM and CBE) with and without synthetics data generation.

#### 3.3.1 Single Variable Distribution Matching Method

The CBE method applied to only a single variable (SVDM) often performs better than the CBE method applied to multiple variables, according to the MAE criteria. The likely reason is that a better match can be made for the most important variable when variables of minor importance to the damage prediction are not considered for determining the weight, and including all variables in the CBE method leads to the best performance on the MBE criteria compared to SVDM.

In practice, a transfer will often need to be made over several important variables. For future research, multiple variables could be used to determine the weights for the training data. In this way, a balance needs to be created between not diluting the influence of the most important variables on the weight, and correcting for biases in multiple variables rather than one. In addition, the user needs to determine whether absolute or average errors are most important for the application of the model.

#### 3.3.2 Synthetic Data Generation Method

The synthetic data generation combined with a sample selection bias correction method generally performs better than just the sample selection bias correction. This is especially the case for the MAE evaluation criteria. The reason this works is probably because ML methods can create very sharp decision boundaries. This means that when a few data points have very large weights the ML models can infer that only under the specific conditions of these data points the related high damage occurs but not with similar values. For example, according to the model, a large damage could only occur at 4 m water depth but not at 3.9 m or 4.1 m. This is a form of overfitting. The synthetic data generation methods introduce some variation in these high weighted samples and hence increase the decision region for which the ML method assigns a high damage. This is the same reason why the similar SMOTE method performs well (Chawla et al., 2002).

The disadvantage of the synthetic data generation methods is that information inside the data might be lost while building the statistical model to draw synthetic data points. A future method would be desirable that also increases the decision region but minimizes the loss of information from the original data. A possible approach that could be considered is the use of differential privacy techniques (Khatri, 2017). These techniques add small perturbations to the data to reduce privacy concerns. Recently, Khatri (2017) found that these perturbations work to prevent overfitting also.

## 4 CONCLUSIONS

Recent advances in damage models include data‐driven methods to estimate damages caused by natural hazards. An important quality of such methods is their ability to capture complex, nonlinear relationships between multiple variables related to hazard, exposure, and vulnerability. However, data‐driven methods are usually limited by the availability and quality of the data required to build such models. As a result, transfer of the models (i.e., using data from one location to build a model for another location) is often required. This raises a problem, the sample selection bias, as the collected data are often not fully representative for the situation it needs to be applied on.

This study was undertaken to improve such methods to correct for this sample selection bias, and to evaluate the quality of the predictions. Such corrections were applied on two different case studies: (i) a macrolevel damage model for typhoons in the Philippines and (ii) a microlevel damage model for European river flood damages.

Two ML techniques were used: RFs and ANNs. They were then improved by using the three different methods to correct the sample selection bias: KMM, CBE, SVDM, which apply weights to the training data. As sometimes very high weights are assigned to specific observations, additionally, a statistical model was built to generate a larger set of synthetic training data before the ML techniques were applied.

We conclude that multivariable data‐driven damage models should correct for the sample selection bias that arises from a model transfer setting, as especially on the MBE large reductions are possible, amount to more than 30% error reduction. For a large model transfer (e.g., data from small typhoon to predict damages from an extreme typhoon), the ANN method seems to further improve the predictions compared to the RF method, probably because the method is better capable of extrapolation. These sample selection bias correction methods are especially important in reducing MBEs for the micromodels and lead to up to 50% reduction on MBE, compared to reductions up to 10% on the MAE. For macromodels the correction methods are shown to also reduce the MAEs, with a reduction up to 20%.

Synthetic data points generated from the sample selection bias correction methods are shown to considerably improve the models for the MAE criteria, and more than half of the improvement is introduced by the synthetic data for the MAE metric. Future studies that correct for a sample selection bias should therefore consider extending the data set using synthetic data generation after the sample selection bias correction.

This study shows that in the future, data‐driven damage models should consider sample selection bias correction methods when a model transfer is required. This helps to reduce the MBE and to better predict outlier observations. To correctly predict these outlier cases synthetic data generation or similar techniques can be used. In transfer cases where the simulation of extreme values beyond the observational data is required, ML techniques should be considered that can allow extrapolation, such as ANN in this study.

Further research could help establish a reliable impact‐based forecasting system based on data‐driven multivariable models. This system would be of great help for several sectors, ranging from insurance industry to humanitarian aid organizations. The insurance industry can apply this model to estimate risk premiums. Humanitarian organizations can use data‐driven predictions to prioritize faster and better their preparation and aid distribution process in the early warning /early action phase, and after a disaster strikes.
